# Exploring the link between parents’ differentiation of self and children’s externalizing behavior problems: the mediating role of need-supportive vs. need-frustrating parenting practices

**DOI:** 10.3389/fpsyg.2024.1387944

**Published:** 2024-08-12

**Authors:** Michal Klein, Tomer Levy, Cory Shulman, Etan Lwow, Tamar Silberg

**Affiliations:** ^1^School of Social Work and Social Welfare, The Hebrew University of Jerusalem, Jerusalem, Israel; ^2^Behavior Regulation Service, Geha Mental Health Center, Petah Tikva, Israel; ^3^Department of Social Work, Tel-Hai Academic College, Upper Galilee, Israel; ^4^Faculty of Medicine, Tel Aviv University, Tel Aviv, Israel; ^5^Ayeka-The Developmental Bond, Development and Parenting Center, Kfar Saba, Israel; ^6^Department of Psychology, Bar-Ilan University, Ramat-Gan, Israel; ^7^Department of Pediatric Rehabilitation, Edmond and Lily Safra Children's Hospital, Sheba Medical Center, Ramat-Gan, Israel

**Keywords:** parental stress, emotional regulation, self-determination theory, child development, gender differences, family systems theory

## Abstract

**Objective:**

Externalizing behavior problems (EBPs) are common in children, with significant long-term impact on the child and family members. Parents, particularly mothers, of children with EBPs often experience heightened emotional distress. One crucial factor affecting parents’ ability to manage this distress is their level of differentiation-of-self (DOS). Differentiated parents are more likely to engage in practices that meet their child’s psychological needs, thus supporting the self-determination theory principles vital for a child’s well-being. This study examined the impact of parental DOS on parenting practices and subsequently on the child’s EBPs, exploring possible differences between mothers and fathers.

**Methods:**

Thirty-two mother–father dyads with children aged 6–14, diagnosed with EBPs participated. Parents completed the Differentiation of Self Inventory–Short Form, the Revised Parents as a Social Context Questionnaire, and the Strengths and Difficulties Questionnaire to assess parental DOS, practices, and child’s EBPs, respectively. Adjusted parallel mediation models examined the mediating role of parental practices in the relationship between parental DOS and a child’s EBPs.

**Results:**

While no direct link between parental DOS and child’s symptoms was found, a complete mediation model indicated need-frustrating practices mediating between parental DOS and a child’s EBPs, for both mothers and fathers. Additionally, fathers’ need-supportive practices, but not mothers’, were negatively associated with the child’s symptoms.

**Discussion:**

These findings highlight the interaction between parental traits, need-frustrating practices, and a child’s psychopathology. Notably, fathers’ supportive behaviors emerged as potential protective factors against child’s EBPs, suggesting promising directions for future research and interventions targeting fathers.

## 1 Introduction

Externalizing behavior problems (EBPs) are among the most prevalent disorders in children under 11 years old, second only to anxiety disorders in youth aged 12–17 years (Polanczyk et al., 2015; Centers for Disease Control and Prevention, 2023) EBPs are more commonly diagnosed in boys compared to girls across all age groups (Ghandour et al., 2019). The literature suggests that EBPs are characterized by outward-directed behaviors such as aggression, impulsivity, defiance, hostility, and difficulties with impulse control (Achenbach et al., 2016). Furthermore, EBPs can significantly impact a child’s development and well-being (Erskine et al., 2016; Fairchild et al., 2019) with a higher risk of developing anxiety and depression disorders (Ghandour et al., 2019). Children with EBPs are also at an increased risk for long-term academic and occupational difficulties, financial problems, challenges in relationships, mental health issues, criminal behavior, and high-risk sexual behavior (Colman et al., 2009; Erskine et al., 2016; Fairchild et al., 2019). This group of disorders can have far-reaching negative consequences that impact not only the child in adulthood but also the entire family (Parsonage, 2009; Smith et al., 2010; Bonin et al., 2011; Zhao et al., 2019).

Studies consistently show that parents of children with EBPs report higher levels of stress (Solem et al., 2011; Theule et al., 2013; Wiener et al., 2016; Mikolajczak et al., 2018; Tokunaga et al., 2019;); the severity of their child’s EBPs directly correlates with parents’ self-reported stress, serving as a proxy measure of their psychological burden (Vierhaus et al., 2013; McSherry et al., 2018). With regard to child-related parental stress, research suggests that mothers often experience higher stress levels linked to their child’s EBPs than do fathers (Dabrowska-Zimakowska and Pisula, 2010; McStay et al., 2014; Calvano et al., 2022). High-level parental stress indicative of relational strains between parent and child, is known to correlate with adverse parents’ mental and physical health (Eisenhower et al., 2009).

These findings further complicate this relationship since parental stress can also diminish the effectiveness of parenting practices (Anthony et al., 2005; Pimentel et al., 2011; Barroso et al., 2018; Levy et al., 2023), potentially creating a self-perpetuating cycle of parental stress and child’s behavioral difficulties.

The development of EBPs in children is complex and influenced by multiple factors (Fairchild et al., 2019; Hawes et al., 2023). A large body of empirical data highlights the role of parenting styles and behaviors in children’s EBPs. Studies have identified harsh, coercive, dysregulated, inconsistent, and warmth-depleted parenting practices as significant risk factors for the development of EBPs during childhood (DeGarmo et al., 2004; Waller et al., 2015; Fairchild et al., 2019). The leading to the growing use of parent-based therapies as a central approach in treating children with EBPs (Daley et al., 2018; Kazdin et al., 2018; Allen et al., 2019; Ghandour et al., 2019). Such interventions demonstrate the potential for improving children’s behavior by working directly with their parents, even without directly targeting the child (Daley et al., 2018; Fairchild et al., 2019; Ghandour et al., 2019).

Recent studies have continued to support the importance of parenting styles within contemporary parenting frameworks (Sarwar, 2016; Pinquart, 2017; Smetana, 2017; Kuppens and Ceulemans, 2019). A systematic review and meta-analysis by Pinquart (2017) underscored the long-term developmental effects of parenting styles on children’s EBPs. Authoritative parenting was found to have a modest negative association with EBPs over time, while authoritarian and neglectful parenting had stronger positive associations. These findings, along with other studies, highlight the ongoing relevance of parenting styles for the current understanding of child behavioral adjustment and the development of targeted intervention efforts, particularly in the context of addressing parental effects on EBP symptomatology in children.

The complex interplay between parents’ characteristics and behaviors and a child’s well-being has long been the subject of developmental research. Self-Determination Theory (SDT), proposed by Deci and Ryan (2000), offers a framework for understanding this relationship. According to SDT, three fundamental psychological needs – autonomy, competence, and relatedness – are essential for human motivation and growth. Specifically, parenting practices that support these needs, characterized by warmth, creating a structured environment, and fostering autonomy, are associated with improved child adaptation and enhanced mental well-being (Joussemet et al., 2005; Soenens and Vansteenkiste, 2010; Allen et al., 2019). Conversely, parenting practices that frustrate these basic needs, characterized by rejection, creating a chaotic and unclear environment and using coercive practices, have been correlated with higher levels of emotional and behavioral problems in children and adolescents (Skinner et al., 2005; Liga et al., 2017; Barberis et al., 2023). Research has suggested gender differences in parenting practices (Yaffe, 2023), with mothers generally exhibiting more warmth and fathers perceived as employing more authoritative approaches (Endendijk et al., 2016; Yaffe, 2023). To the best of our knowledge, there is a paucity of research about the differential impacts of need-supportive versus need-frustrating practices exhibited by fathers and mothers on child outcomes.

While research has identified specific parenting practices that contribute to child EBPs (Barker et al., 2012; Jami et al., 2021), less is known about the underlying parental characteristics that shape these behaviors. The concept of differentiation of self (DOS) from family systems theory provides a useful framework for understanding this link. DOS is perceived as a central concept associated with parents’ ability to regulate their emotional state (Endendijk et al., 2016; Yaffe, 2023). According to the literature, DOS consists of four characteristics: fusion with others, emotional reactivity, I-position, and emotional cut-off (Bowen, 1966; Titelman, 2014). The level of parental DOS has a crucial role in shaping parenting practices and subsequent child outcomes. Specifically, *fusion with others* refers to the extent to which an individual feels a need to conform to others’ expectations and struggles to maintain emotional autonomy. Parents with high levels of fusion may be emotionally dependent on their children and feel pressured to cater to their needs, even at the expense of their own (Skowron and Friedlander, 1998; Skowron and Schmitt, 2003; Klever, 2005). *Emotional reactivity* describes the tendency to respond automatically and impulsively to emotional stimuli, rather than in a thoughtful and regulated manner (Titelman, 2014; Yavuz Güler and Karaca, 2021). Parents with high emotional reactivity may find it challenging to maintain emotional stability and to contain their child’s emotions (Skowron et al., 2010; Titelman, 2014). An *I-position* reflects the ability to clearly express one’s own thoughts, feelings, and beliefs, even in situations of conflict or pressure from the environment. Parents with a strong I-position are capable of making decisions and acting according to their values, even when faced with resistance from their child (Skowron and Friedlander, 1998; Titelman, 2014). Lastly, *emotional cut-off* refers to the propensity to disconnect or withdraw from relationships when coping with emotional tensions. Parents prone to emotional cut-off may distance themselves from their children during times of distress instead of offering support and empathy (Titelman, 2003; Knauth and Skowron, 2004). These four components of DOS have been linked to parents’ ability to regulate their emotions and respond appropriately to their children’s needs (Skowron and Friedlander, 1998; Skowron and Schmitt, 2003). Parents with higher levels of DOS demonstrate better emotional regulation, not only within themselves but also in relation to their children (Papero, 2014). Moreover, studies have found a connection between parental DOS and the child’s symptoms and psychological adjustment, with children of parents with lower DOS experiencing more emotional distress and behavioral problems (Skowron, 2005; Skowron et al., 2010; Peleg et al., 2014; Yavuz Güler and Karaca, 2021; Simon and Scher, 2023;).

According to family systems theory and the DOS concept (Barker et al., 2012), higher levels of DOS are theorized to facilitate need-supportive practices (e.g., autonomy support, warmth) and optimal child adjustment, while lower differentiation may promote need-frustrating behaviors (e.g., coercion, rejection) linked to child maladjustment (Knauth and Skowron, 2004; Egeli et al., 2015). However, to our best knowledge, direct empirical research has yet to examine these associations between DOS and specific parenting behaviors.

Given the high burden and stress experienced by parents of children with EBPs, understanding parental factors like DOS that may shape their parenting practices and subsequently impact child EBPs is crucial for developing targeted, parent-focused interventions. However, existing literature suggests potential gender differences in parental DOS and its manifestations (Peleg, 2008; Drake, 2011; Shwalb et al., 2013; Fagan et al., 2014; Simon and Scher, 2023). Fathers are becoming more similar to mothers in terms of their parenting roles and time spent with children, at least in Western societies (Shwalb et al., 2013; Panter-Brick et al., 2014). Yet the evidence regarding fathers’ and mothers’ differential contributions to child development and adjustment remains limited and inconclusive, partly due to the lack of research directly comparing them (Fagan et al., 2014). Therefore, examining the proposed associations parental DOS, parenting practices, and children’s EBPs separately for mothers and fathers is required for a more nuanced and complete understanding of the distinct roles mothers and fathers may have in children’s behavior. Elucidating these potentially differential effects can inform the development of parent-focused interventions that are tailored to the specific needs and contributions of mothers and fathers.

### 1.1 Research aims and hypotheses

**Figure 1:**
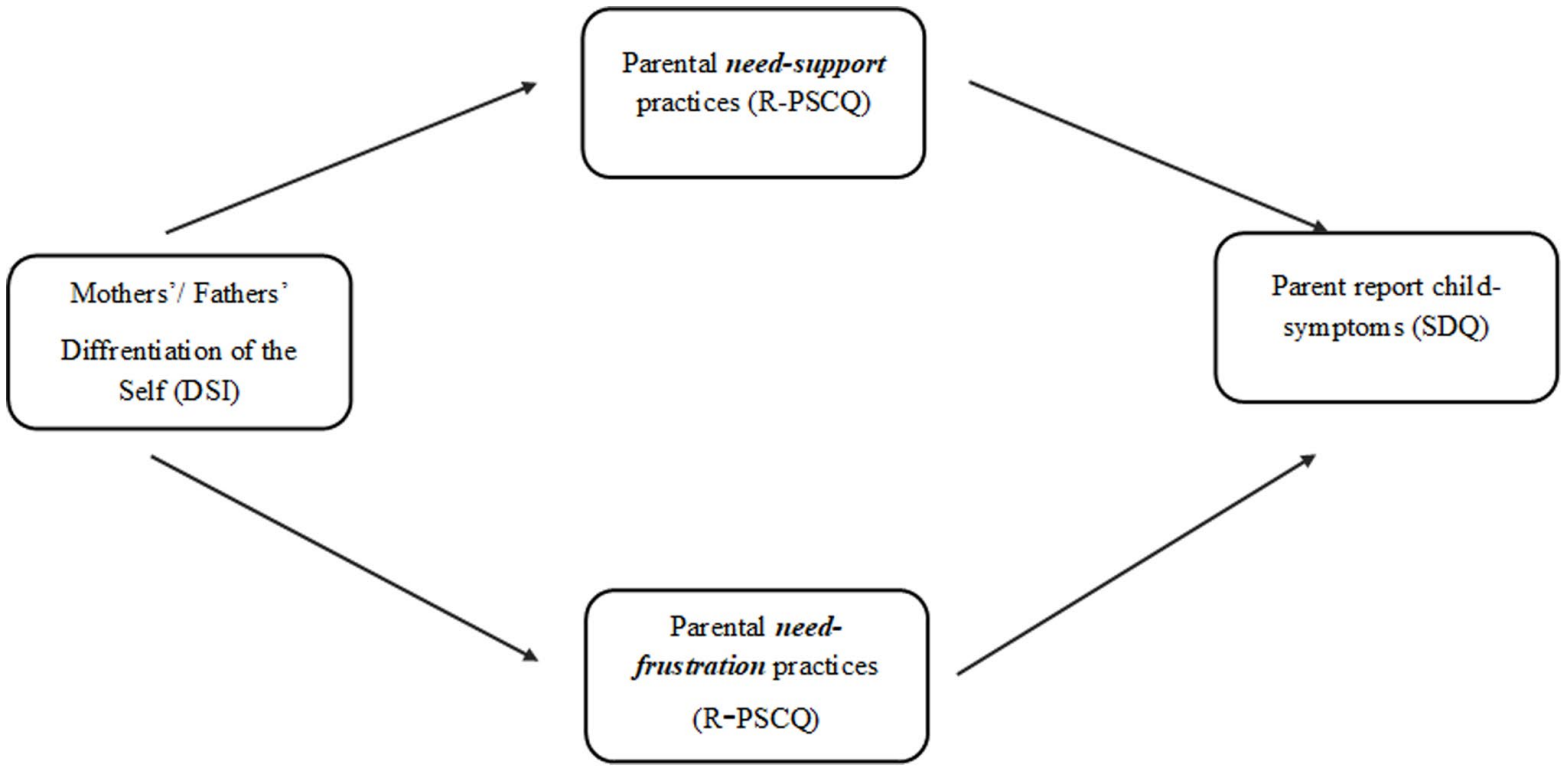
The theoretical model of the mediating role of parental practices (need-support vs. need-frustrate) in the association between mothers’ and fathers differentiation of the self and parent report child-symptoms.

This study aimed to examine the associations between parental DOS, parenting practices (supporting and frustrating a child’s basic psychological needs), and the severity of a child’s EBPs, focusing on potential differences between mothers and fathers. We hypothesized that: (1) lower levels of parental DOS would be associated with the child’s EBPs; (2) lower levels of parental DOS would be associated with more need-frustrating parenting practices; (3) higher levels of parental DOS would be associated with more supportive parenting practices; (4) need-frustrating parenting practices would be positively associated with a child’s EBPs; (5) supportive parenting practices would be negatively associated with a child’s EBPs; and (6) parenting practices (both supportive and frustrating) would mediate the relationship between parental DOS and the child’s EBPs (Figure 1).

Due to the inconsistency in the literature regarding gender differences in parental DOS and practices, all hypotheses were also addressed separately in the results section to assure that they support the executed analysis.

## 2 Methods

### 2.1 Study design

The current sample is part of a larger study examining a parent-based intervention for children with EBPs, using the AYEKA protocol (Cohen and Lwow, 2004). The current analysis is based on data collected at baseline during the pre-intervention phase. Baseline data included initial screening performed by the research staff at the clinic.

Recruitment was conducted at a tertiary hospital clinic specializing in EBPs. Initial screening was performed by the clinic’s professional staff based on data collected during the routine intake and evaluation process. All patients meeting the inclusion criteria were asked to participate in the study.

Inclusion criteria were: (1) parents of a child aged 6–14 years diagnosed with EBP by a certified psychiatrist or psychologist from the clinic; (2) consent of the child’s guardians to participate in the study; and (3) a reasonable level of spoken Hebrew to be able to complete questionnaires and follow research protocol. Exclusion criteria were: (1) children with intellectual developmental disability, autism spectrum disorder, previous suicide attempts, bipolar disorder, psychosis, post-traumatic stress disorder, neglect and/or abuse; and (2) parents diagnosed with intellectual developmental disability, autism spectrum disorder, depression, bipolar disorder, schizophrenia, active psychosis, personality disorder, post-traumatic stress disorder or involved in an active conflict.

A child and adolescent psychiatrist determined the clinical diagnoses as part of the routine evaluation process.

### 2.2 Participants

Participants were 34 dyads of parents of children aged 6–14 years (M = 10.57, SD = 2.50, 82.4% boys) diagnosed with at least one or more of the following disorders: Attention-Deficit/Hyperactivity Disorder, Oppositional Defiant Disorder, Intermittent Explosive Disorder, Disruptive Mood Dysregulation Disorder, and/or Conduct Disorder. Among these dyads, 30 (88.2%) were cohabiting, while four (11.8%) were living separately (separated or divorced).

Table 1 presents the sociodemographic characteristics of the parent sample. The current study focuses on pre-intervention quantitative baseline measures (Time 0). The study received approval from the Helsinki Committee of the mental health center (approval no. 0015-21-GEH).

**Table 1:** Background characteristics of the mothers and fathers of children with EBPs.

Background characteristics	Values	Frequency
Parent status	Cohabiting	30 (88.2%)
	Living separately	4 (11.8%)
Mother’s educational level	Elementary School	1 (2.9%)
	High School	7 (20.6%)
	BA/Professional certificate	13 (38.2%)
	Master’s degree or higher	13 (38.2%)
Father’s educational level	Elementary School	0 (0.0%)
	High School	7 (20.6%)
	BA/Professional certificate/BA	22 (64.7%)
	Master’s degree or higher	5 (14.7%)
Mother’s recent financial loss	No	7 (20.6%)
	Yes	27 (79.4%)
Father’s recent financial loss	No	6 (17.6%)
	Yes	28 (86.4%)
Child’s gender	Boy	28 (82.4%)
	Girl	6 (17.6%)
Child’s medication consumption	No	23 (67.6%)
	Yes	11 (32.4%)

### 2.3 Measures

Children’s Externalizing Behavior Problems (EBPs) were measured using the parent-reported Strengths and Difficulties Questionnaire (SDQ; Goodman, 1997). The SDQ contains four psychopathology subscales: (1) emotional symptoms, (2) behavioral/conduct problems, (3) hyperactivity problems, and (4) peer problems, along with one prosocial functioning subscale. Each subscale consists of five items and is rated on a 0 to 2 Likert-type scale (0 = never true, 1 = sometimes or somewhat true, and 2 = very true or very often true). The total score is computed using the four psychopathology subscales, with higher scores indicating higher levels of behavioral problems. Internal consistency for both mothers and fathers in the current study was good [Cronbach’s α = 0.84 and α = 0.82, respectively].

Parental Differentiation of Self (DOS) was assessed using the Differentiation of Self Inventory–Short Form (DSI-SF; Drake, 2011), a well-validated measure aligned with Bowen’s family systems theory and widely used in prior research on parental alienation (Skowron and Friedlander, 1998; Skowron and Schmitt, 2003). The DSI-SF is based on the Differentiation of Self Inventory – Revised (DSI-R; Skowron and Schmitt, 2003) and was translated into Hebrew by Peleg (2008). The DSI-SF is comprised of 20 items rated on a Likert scale ranging from 1 = “not at all true for me” to 6 = “very true for me.” It encompasses four factors consistent with the self-differentiation theory: (1) fusion with others (five items), (2) emotional reactivity (six items), (3) I-Position (six items), and (4) emotional cutoff (three items). Internal consistency for both mothers and fathers in the current study was high [α = 0.84 and α = 0.89, respectively].

Parenting practices were measured using the Revised Parents as a Social Context Questionnaire (R-PSCQ; Skinner et al., 2005). The R-PSCQ is a comprehensive measure that assesses both supportive and frustrating child’s psychological needs in parenting practices, consistent with the study’s theoretical framework grounded in Self-Determination Theory (Deci and Ryan, 2000; Joussemet et al., 2005). The R-PSCQ is designed for parents of children aged 2–18 years, and measures six parenting dimensions: (1) Autonomy Support, (2) Coercion, (3) Structure, (4) Chaos, (5) Warmth, and (6) Rejection. Each dimension contains six items rated on a 7-point Likert scale ranging from 1 = “completely disagree” to 7 = “completely agree.” Previous studies have reported good construct validity and internal reliability for the six subscales, with Cronbach’s alphas ranging from α = 0.61 to α = 0.82 (Allen et al., 2019; Rebecka et al., 2020). Aligned with SDT, the three positive dimensions of parental behaviors (warmth, structure, and autonomy support) were merged into a single scale reflecting parenting practices that support a child’s psychological needs. The three negative dimensions (rejection, chaos, and coercion) were combined into a separate scale representing parenting practices that frustrate a child’s psychological needs (Skinner et al., 2005; Egeli et al., 2015; Rebecka et al., 2020). An average calculation of each scale was employed based on the professional literature (Skinner et al., 2005; Rebecka et al., 2020). The English version was translated into Hebrew with permission from the author using the back-translation method. Two translators (native English and Hebrew speakers) worked collaboratively until reaching consensus. The translated version was then back-translated into English. The internal consistency for the 15 items which measure parenting practices that support a child’s psychological needs for both mothers and fathers was high [α = 0.76 and α = 0.87, respectively] as were the 15 items which measure parenting practices that frustrate a child’s psychological needs [α = 0.85 and α = 0.87, respectively].

### 2.4 Data analyses

Data were analyzed using SPSS.29 and R software. Descriptive analyses (distribution, means, and standard deviations) of each of the quantitative study variables among all participants were performed for the background variables. To examine whether parental levels of DOS, parenting practices, and a child’s EBPs differ between mothers and fathers, paired samples t-tests were conducted. Pearson correlation tests were also conducted to examine the association between mother’s self-report and father’s self-report on the various study measures. Correlations and hierarchical regression analyses were used to test hypotheses 1–5.

In addition, multilevel modeling (MLM) analyses were conducted using the ‘lme4’ package in R to account for the nested structure of the data (i.e., parents nested within families). MLM allows for the examination of both within-family and between-family effects while controlling for the non-independence of observations. The ICC (intraclass correlation coefficient) was calculated to assess the proportion of variance in the outcome variables attributable to differences between families.

Hayes’s (2013) PROCESS macro using 5,000 bootstrap resampling for calculation of confidence intervals was used to test hypotheses 6–7 [mediator model 4; for the advantages of using this macro, see Hayes, 2009].

## 3 Results

### 3.1 Differences between mothers and fathers

The results indicated that parental levels of DOS, parenting practices, and the child’s EBPs did not differ significantly between mothers and fathers. However, within the specific factors of parental DOS, significant differences were found between mothers’ and fathers’ emotional cutoff scores [*t*(32) = 2.64, *p* < 0.05], with mothers reporting higher levels of emotional cutoff (M = 14.88; SD = 3.98) compared to fathers (M = 13.09; SD = 4.37). In addition, significantly high positive correlations were found between mothers’ self-report and fathers’ self-report on children’s EBPs [SDQ; *r*(32) = 0.70, *p* < 0.001]. Of the parental variables, significant positive correlations were found between mothers’ and fathers’ reports on their tendency to react in emotional cutoff [*r*(32) = 0.55, *p* < 0.01], and their use of warm [*r*(32) = 0.472, *p* < 0.001] and chaotic practices [*r*(32) = 0.35, *p* < 0.05; see Table 2].

**Table 2:** Mean, SD and *F*-values of mothers’ and fathers’ levels of differentiation, parental practices, and parent reported child-symptoms.

	Correlation coefficient^1^	Mothers		Fathers		*T*-values		
Study variables		Mean	SD	Mean	SD	*t*	*p*	Cohen’s d
Parental levels of differentiation								
Differentiation of the self	0.11	85.88	14.55	85.24	17.08	0.18	0.860	0.03
Emotional reaction	0.10	20.97	6.88	23.09	7.21	1.30	0.202	0.22
I position	0.03	27.41	4.34	26.97	5.16	0.39	0.700	0.07
Emotional cut-off	0.55^***^	14.88	3.98	13.09	4.37	2.64^*^	0.013	0.45
Fusion with others	0.18	22.62	4.38	22.09	5.30	0.49	0.624	0.08
Parenting practices								
Supportive of child’s needs	0.36	3.41	292.	3.34	396.	1.57	0.127	0.27
Warmth	47.^**^	3.31	391.	3.27	513.	0.25	0.61	0.00
Structure	76.-	3.48	412.	3.45	401.	0.08	0.77	0.00
Autonomy support	31.	2.77	598.	2.61	574.	1.89	0.17	0.05
Frustration of child’s needs	0.25	2.30	495.	2.30	534.	0.18	0.857	0.03
Rejection	06.	2.33	391.	2.33	694.	0.00	1.00	0.00
Chaos	^*^35.	1.80	568.	1.94	606.	1.38	0.24	0.04
Coercion	0.27	2.77	598.	2.61	574.	1.30	0.26	0.03
Parent report child-symptoms								
SDQ score	0.70^***^	18.17	6.98	18.64	6.97	0.51	0.615	0.09

SDQ = strengths and difficulties questionnaire.

1 The correlation coefficients between the mother’s reports and the father’s reports in the various research measures.

* *p* < 0.05.

*** *p* < 0.001.

### 3.2 Correlations between parental differentiation of the self, parenting practices and the child’s EBPs

The results indicated that contrary to our first hypothesis, no correlation was found between mothers’ and fathers’ DOS and children’s EBPs [*r*(32) = −0.12, *p* = 0.481 and *r*(32) = −0.16, *p* = 0.380, respectively]. Furthermore, and in contrast to our second hypothesis, no significant correlation was observed between both mothers’ and fathers’ levels of DOS and parenting practices that support psychological needs [*r*(32) = −0.05, *p* = 0.794 and *r*(32) = 0.21, *p* = 0.227, respectively]. In accordance with our third hypothesis, levels of DOS of both mothers and fathers were negatively associated with parenting practices that frustrate psychological needs [*r*(32) = −0.50, *p* = 0.003 and *r*(32) = −0.44, *p* = 0.009, respectively]. In addition, our fourth hypothesis was partially supported, with parenting practices that support psychological needs and a child’s EBPs negatively correlated among fathers but not among mothers [*r*(32) = −0.37, *p* = 0.031 and *r*(32) = −0.07, *p* = 0.683, respectively]. Lastly, in accordance with our fifth hypothesis, parenting practices that frustrate psychological needs positively correlated with a child’s EBPs among both mothers and fathers [*r*(32) = 0.49, *p* = 0.003 and *r*(32) = 0.49, *p* = 0.003, respectively] (see Table 3).

**Table 3:** Pearson correlation coefficients between parental levels of differentiation, parental practices, and parent report child-symptoms among mothers and fathers of children with EBPs.

	Mothers			Fathers		
	Need-supportive	Need-frustration	Differentiation of the self	Need-supportive	Need-frustration	Differentiation of the self
Parent report SDQ	−0.07	0.49^**^	−0.12	−0.37^*^	0.49^**^	−0.16
Need-supportive		−0.42^*^	−0.05		−0.40^*^	0.21
Need-frustration			−0.50^**^			−0.44^**^

SDQ = strengths and difficulties questionnaire; EBPs = Externalizing behavior problems.

* *p* < 0.05.

** *p* < 0.01.

### 3.3 The mediating role of parenting practices in the association between parents’ differentiation of the self and children’s EBPs

In order to examine our mediation model hypotheses (6 and 7), Hierarchical regression analyses were first conducted for mothers and fathers separately. Background characteristics were entered in the first block of the regression in a stepwise manner. Subsequently, only background characteristics which contributed significantly to the Explained Variance (EPV) were entered into the regression model. These variables were entered in the first block to control for their potential effect on the child’s EBPs (dependent variable; DV). DOS (the independent variables; IV) was entered in the second block, and parenting practices that satisfy or frustrate psychological needs were entered only in the third block to examine whether these variables have a unique contribution to the EPV of the DV beyond that of the DOS (Table 4).

**Table 4:** Results of hierarchical regression analyses for the parent report child-symptoms – SDQ score among mothers and fathers of children with EBPs.

Block	Explanatory variable	*B*	*SE.B*	*β*	*R^2^*	*∆R^2^*
Mothers						
1	Medicated^1^	−31.38	12.19	−0.43^*^	0.181^*^	----
3	Medicated^1^	−33.53	10.38	−0.45^**^		
	Frustration of child’s needs	2.44	0.69	0.50^***^	0.428^***^	0.247^***^
Fathers						
3	Frustration of child’s needs	1.96	0.72	0.45^**^	0.200^**^	----

SDQ = strengths and difficulties questionnaire.

1 Medicated: 0 = The child is not medicated, 1 = The child is medicated.

* *p* < 0.05.

** *p* < 0.01.

*** *p* < 0.001.

Of the many background variables examined, including parental age, child’s age, child’s gender, parents’ marital status, education, job loss, contact with welfare agencies, and child’s stimulant medication treatment, only the child’s medication status had a significant contribution (18.1%) to the explained variance in a child’s EBPs according to mothers’ reports. In the multilevel modeling (MLM) analysis, none of the background variables were found to be significant predictors, although child’s medication status approached significance. The negative β coefficient indicated that mothers reported higher levels of child symptoms when their child was not receiving stimulant medication. No significant contribution of any other background characteristics was found among fathers of children with EBPs. In addition, both mothers’ and fathers’ DOS did not contribute significantly to the level of a child’s EBPs, beyond background characteristics. Finally, only parenting practices that frustrate a child’s psychological needs had a unique contribution to the EPV of child symptoms for both parents. The positive β coefficients indicate that as mothers and fathers reported a higher degree of parenting practices that frustrate a child’s psychological needs, their reports on their child’s EBPs were also more frequent.

Multilevel Modeling (MLM) is a powerful statistical technique for analyzing dyadic data (Jenkins et al., 2009; Ledermann and Kenny, 2017). MLM allows for the examination of relationships between variables while accounting for the nested structure of the data (individuals nested within dyads). In this study, we utilized MLM analysis using the package lme4 in R to examine how parental psychological characteristics (DOS), and their use of need-supportive and need-frustrating parenting practices relate to the severity of a child’s symptoms. The Intraclass Correlation Coefficient (ICC) in the current study indicates substantial variation in the outcome variable within and between dyads (0.7 and above, Model 0). Consistent with previous regression analysis, in the MLM analysis, we entered the parents’ background characteristics, their DOS, and their parenting practices that satisfy or frustrate psychological needs as explanatory variables (Model 1). The results indicated similar findings to the regression analysis previously conducted, suggesting that only parenting practices that frustrate a child’s psychological needs had a unique contribution to the explained variance of child symptoms [*B* = 35.36, *SE.B* = 6.36, *t* = 5.56, *p* < 0.001], even after accounting for the nested structure of the data. Finally, a significant difference between Model 0 (AIC = 675.50, only the variation in the dependent variable) and Model 1 (AIC = 639.00, with the explanatory variables) was found (*χ*^2^ = 34.46, *p* < 0.001). This result confirms that parenting practices that frustrate a child’s psychological needs contribute to explaining child symptoms beyond the nested nature of the dyad data.

**Figure 2:**
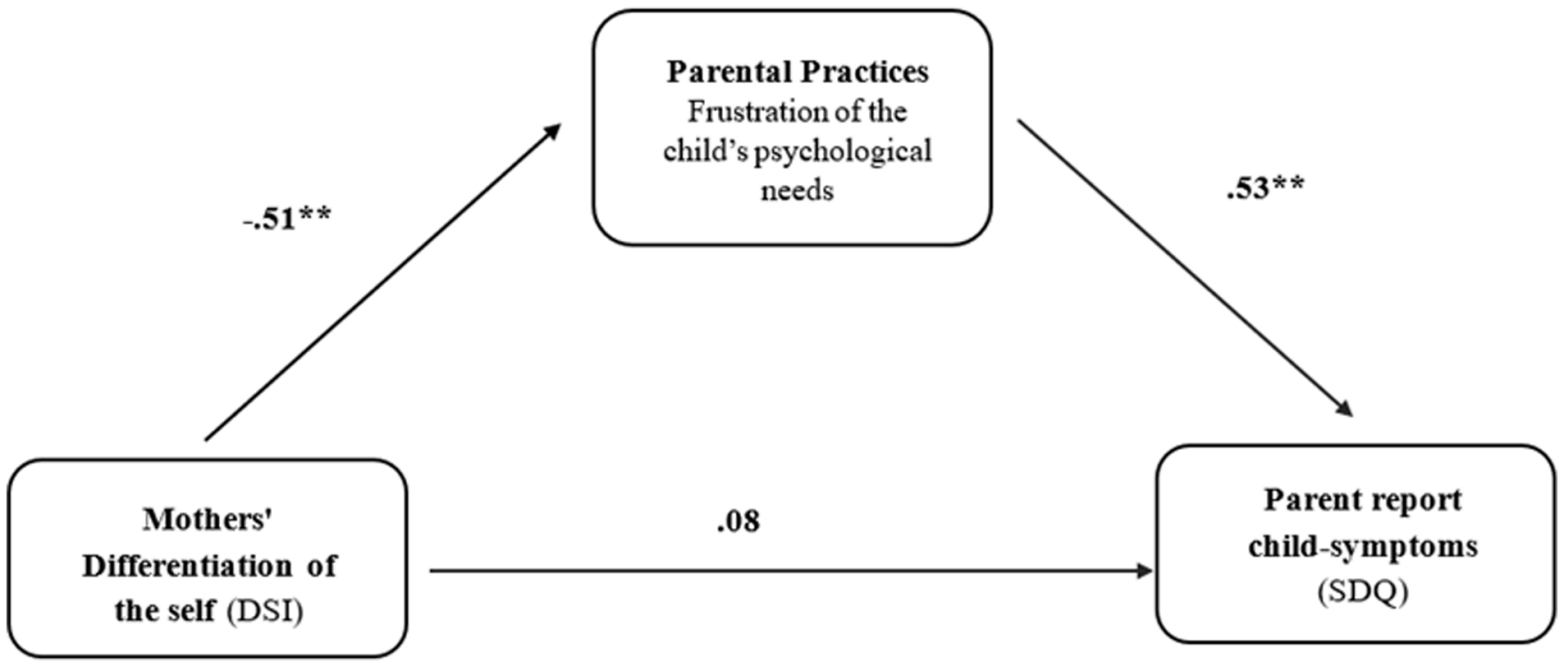
Parenting practices that frustrate a child’s psychological needs as a mediating variable between mothers’ differentiation of the self and parent report child-symptoms controlling over the medication variable among mothers of children with EBPs.

**Figure 3:**
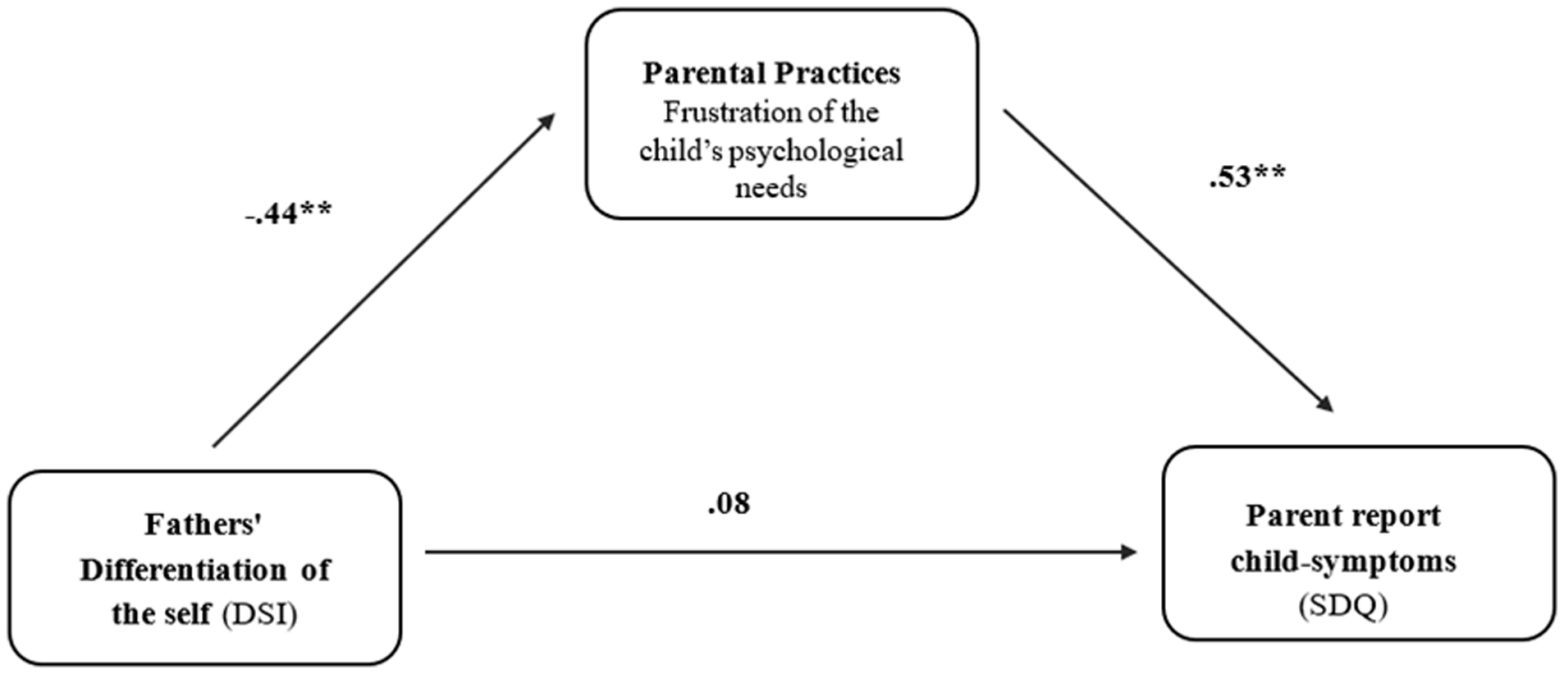
Parenting practices that frustrate a child’s psychological needs as a mediating variable between fathers’ differentiation of the self and parent report child-symptoms among fathers of children with EBPs.

Following the regression and MLM results, a mediation analysis was conducted to examine whether the correlation between mothers and fathers’ DOS and child’s EBPs was mediated by parenting practices that frustrate a child’s psychological needs. The mediation analysis results indicated a full mediation model, with parenting practices that frustrate a child’s psychological needs serving as a significant mediation variable in the association between both mothers and fathers DOS and the child’s EBPs [mothers: 95% CI −0.49: −0.07 and fathers: 95% CI −0.46: −0.05; see Figures 2, 3].

## 4 Discussion

Maladaptive parenting characteristics are recognized as contributing factors to EBPs in children and adolescents (Skinner et al., 2005; Joussemet et al., 2008; Soenens and Vansteenkiste, 2010; Allen et al., 2019; Jami et al., 2021). However, limited data exist on specific dispositional psychological traits of parents that may lead to these negative behaviors. To address this gap in knowledge, the current study investigated whether DOS in parents of children with EBPs is associated with their use of need-supportive and/or need-frustrating parenting practices, and whether these practices are associated with their child’s symptoms. We also wanted to examine whether these associations differ between mothers and fathers.

Contrary to our initial hypotheses, no direct association was found between parents’ DOS and a child’s EBPs. However, our results suggested an indirect pathway, with need-frustrating parenting practices mediating the relationship between parental DOS and a child’s EBPs. Also, our results revealed no discernible disparity between mothers and fathers within the mediation model. The multilevel modeling (MLM) analysis accounting for the nested structure of the data (i.e., parents nested within families) yielded similar results, confirming the robustness of the findings and the importance of targeting need-frustrating parenting practices in interventions aimed at reducing child EBPs. This finding, explored further in subsequent sections, elucidates the potential factors underlying intergenerational transmission of parental stress and the child’s behavioral difficulties.

Our hypotheses regarding the associations between parents’ DOS and their parental practices was partially supported. We found that among both mothers and fathers, levels of DOS were negatively associated with need-frustrating parenting practices. However, no significant associations were found between parental levels of DOS and supportive parenting practices. A possible explanation for the lack of association between parents’ DOS and supportive parenting practices, as opposed to frustrative practices, could be associated with the relatively high levels of prolonged, chronic, and overwhelming parental stress reported among parents of children with EBPs (Wiener et al., 2016; Mikolajczak et al., 2018; Tokunaga et al., 2019). These may have led to parental burnout and the potential erosion of the parent–child relationship (Mikolajczak et al., 2019; Mikolajczak and Roskam, 2020). Parental burnout is characterized by emotional distancing from children, difficulty understanding their developmental needs, and challenges in creating a structured family environment (Mikolajczak and Roskam, 2020; Prikhidko and Swank, 2020). Further research is needed to confirm the detrimental impact of burnout on potential parental risk and resilience factors associated with a child’s EBPs.

Interestingly, our results revealed significant positive correlations between mothers’ and fathers’ emotional cutoff scores alongside significant differences in paired t-tests, with mothers reporting higher levels of emotional cutoff, compared to fathers. Emotional cutoff describes a parent’s attempt to cope with unresolved personal issues by distancing themselves emotionally from their child. This can be expressed by physically moving away from the child or by remaining in physical contact with the child but avoiding sensitive emotional responses (Titelman, 2014). In contrast to previous studies (Peleg et al., 2014) our findings suggested that despite the relatively high correlations, mothers of children with EBPs express higher levels of emotional cutoff compared to fathers. To the best of our knowledge, no previous research has reported on similarities or differences in emotional cutoff between mothers and fathers of children with EBPs. Our findings support previous studies reporting that when a mother distances herself emotionally, her behavior harms family functioning and adolescent behaviors (Crandall et al., 2016). This finding also reinforces the documented higher levels of stress and burnout experienced by mothers of children with EBPs (Dabrowska-Zimakowska and Pisula, 2010; McStay et al., 2014; Calvano et al., 2022), where the resulting exhaustion leads mothers to disengage from both their parental responsibilities and their child’s emotional needs (Mikolajczak et al., 2018). Further research should aim at examining possible sources of maternal and paternal emotional cutoff in response to their child’s EBPs.

Our findings can be interpreted through the lens of the gendered emotion regulation framework (Zimmermann and Iwanski, 2014), which suggests that socialization processes and gender role expectations may lead to differences in emotional regulation strategies between males and females. The higher levels of emotional cutoff observed among mothers in our study may reflect the disproportionate emotional burden and burnout experienced by mothers of children with EBPs (Lebert-Charron et al., 2018). This highlights the need for future research to examine the interplay between gender roles, parental stress, and emotional regulation in the context of parenting children with EBPs, as well as the development of gender-sensitive interventions to support both mothers and fathers.

Another important finding in the current study relates to the significant negative correlation observed only among fathers between supportive parenting practices and children’s symptoms. This finding reinforces previous research suggesting that paternal engagement, particularly in providing emotional support and responsive caregiving, is associated with reduced levels of EBPs (Sarkadi et al., 2008; Panter-Brick et al., 2014; Rolon-Arroyo et al., 2018; Pauli et al., 2021). These results also emphasize the importance of engaging fathers in interventions to support children with EBPs and promote positive family dynamics, and highlight the need for targeted interventions tailored to encompass the potential impact of fathers’ engagement on children’s behavioral outcomes. Unfortunately, studies show that fathers’ engagement in parenting programs is low compared to that of mothers (Panter-Brick et al., 2014; Gonzalez et al., 2023). Overall, our findings underscore the importance of adopting a parent-centered approach that recognizes the unique yet distinct contributions of both mothers and fathers in promoting children’s socioemotional well-being (Piotrowska et al., 2017; Gonzalez et al., 2023;). Further research exploring the specific mechanisms underlying the relationship between paternal support for their child’s basic psychological needs and the child’s behavioral outcomes could provide valuable insights for the development of effective interventions targeting paternal engagement in the context of EBPs.

These findings supports the shifting focus in fatherhood research towards the unique contributions of fathers to child development (Fagan et al., 2014; Panter-Brick et al., 2014). The protective role of paternal supportive practices in the context of child EBPs underscores the importance of father involvement and highlights the need for a more nuanced understanding of the differential effects of maternal and paternal parenting practices. This is also consistent with the relatively new concept of “sensitive fathering” (Giannotti et al., 2022; Bakermans-Kranenburg and van IJzendoorn, 2023), which emphasizes the role of paternal responsiveness and emotional support in fostering a child’s well-being. Future research should further investigate the mechanisms through which paternal supportive practices can provide a buffer against child EBPs, and how these processes may differ from maternal influences.

The current findings also revealed that among both mothers and fathers, need-frustrating parenting practices mediated the association between parents’ DOS and child’s symptoms’ severity, while supportive practices did not mediate this relationship. This suggests that the two dimensions of parenting practices, supporting a child’s psychological needs and frustrating a child’s psychological needs, are not necessarily an opposite expression of the same phenomenon and do not operate in an alternating manner (Skinner et al., 2005; Rebecka et al., 2020). For instance, Skinner et al. (2005) found that seemingly supportive parental behaviors may coexist with rejecting behaviors, potentially creating a confusing and unpredictable environment for the child. Similarly, parents who score high on measures of both fulfilling and frustrating their child’s needs may exhibit inconsistent and mixed messaging, leading to an unpredictable child-rearing environment (Skinner et al., 2005).

Our findings highlight the crucial role of parenting practices, particularly those that frustrate a child’s psychological needs, in the relationship between parental DOS and children’s EBPs. The complete mediation model provides a rather unique perspective that differs from previous research by suggesting that instead of focusing solely on parental characteristics, it may be more effective to target specific parenting behaviors in attempting to reduce child EBPs. This shift in focus underscores the importance of developing interventions that prioritize modifying need-frustrating parenting practices.

Additionally, it is important to highlight that the average scores for frustrative parenting practices within the present sample were significantly higher compared to those documented in a previous study among parents of typically developing adolescents (Rebecka et al., 2020). Conversely, the levels of supportive practices remained relatively consistent with the results found in that specific study. Perhaps this suggests that inconsistent parenting practices may be a risk factor in the development of EBPs among children and adolescents.

### 4.1 Limitations

The strengths of this study were the well-characterized clinic sample of children with EBPs, the multiple informant analysis, and the use of standard measures to evaluate both parental characteristics and behaviors. Nonetheless, there were several limitations to the current study. First, the relatively small sample size used in this study may have limited the applicability of the results to the broader population of children with EBPs. Second, the sample included only traditional family structures (father–mother–child) and did not encompass additional family configurations. Furthermore, the fact that the vast majority of the child participants were boys within a specific age range (6–14) also reduces the generalizability of the results. Further research is needed with a larger and more heterogeneous sample. Considering the study methods, the fact that a cross-sectional design was utilized hindered its ability to establish causality between variables. While the report suggests possible pathways between parental traits (such as DOS) and parental behaviors (parenting practices), it cannot definitively claim that DOS leads to specific parenting practices which then impact child symptoms. Moreover, all measures used in the study were self-reported by the parents themselves, which can be susceptible to bias and social desirability effects. However, the fact that for the majority of the study variables no significant correlations were found between mothers and fathers may reduce such risk of bias. Future studies should aim at collecting information from the children themselves as well as from other potential informants (i.e., teachers, clinicians). Finally, the limited exploration of possible moderators may be considered another weakness of the current design. Although our focus was mainly on the interrelations between parental traits and practices associated with the stressful implications of raising a child with EBPs, we did not explore additional potential factors such as parental burden or parental psychopathology possibly associated with DOS, parenting practices and child’s symptoms.

### 4.2 Research implications

The findings of the current study provide important insights for understanding EBPs in children. Theoretically, the results supported the crucial role of parental DOS in shaping parenting practices and child outcomes, sustaining Bowen’s family systems theory (Kerr and Bowen, 1988). The study highlights the importance of considering both parents’ contributions to child development, with fathers’ supportive practices emerging as a potential protective factor against child’s EBPs. These supportive practices may help create a nurturing environment that promotes children’s emotional regulation and reduces the likelihood of developing EBPs. Our results also support the relevance of incorporating concepts associated with SDT, emphasizing a more holistic approach to the treatment of EBPs in children and adolescents (Skinner et al., 2005).

Clinically, our findings indicated the importance of enhancing parents’ self-regulation abilities and reducing need-frustrating behaviors as a central focus in parent-based interventions aimed at reducing a child’s EBPs.

Given the significant impact of these behaviors on child outcomes, clinicians should prioritize discouraging parents from practices known to frustrate a child’s psychological needs, while providing strategies for parents to regulate their own emotions and respond appropriately to their child’s needs. Additionally, it is crucial to involve both mothers and fathers in treatment and tailor interventions to each parent’s unique role.

Lastly, the study points to several future research directions, such as using a longitudinal design to examine the causal links between parental DOS, parenting practices, and children’s EBPs; investigating specific processes and boundary conditions that explain the differential effects of gender on parent and child-related factors; and testing the efficacy of differentiation-based therapies and father-focused components in reducing EBPs and promoting family functioning. Replication studies in diverse populations are needed to assess the generalizability of the findings (Lamela et al., 2016).

In conclusion, the current study makes a significant contribution to understanding the complex interplay between parental DOS, parenting practices, and children’s EBPs, underscoring the importance of a parent-centered approach in research and clinical practice. The findings have the potential to inform the development of more targeted and effective interventions for children with EBPs, ultimately promoting better outcomes for children and families. By focusing on enhancing parental self-regulation, reducing need-frustrating practices, and engaging both mothers and fathers in treatment, clinicians and researchers can work towards more comprehensive and tailored approaches to addressing EBPs in children.

## Data availability statement

The raw data supporting the conclusions of this article will be made available by the authors, without undue reservation.

## Ethics statement

The studies involving humans were approved by the Helsinki Committee of Geha Mental Health Center, Petah Tikva, Israel (approval no. 0015-21-GEH). The studies were conducted in accordance with the local legislation and institutional requirements. The participants provided their written informed consent to participate in this study and all data has been anonymized to protect participant privacy.

## Author contributions

MK: Conceptualization, Data curation, Formal analysis, Methodology, Writing – original draft. TL: Conceptualization, Methodology, Writing – review & editing, Writing – original draft. CS: Conceptualization, Supervision, Writing – review & editing. EL: Writing – review & editing. TS: Conceptualization, Methodology, Supervision, Writing – review & editing.
